# A Comparison of the Energetic Cost of Running in Marathon Racing Shoes

**DOI:** 10.1007/s40279-017-0811-2

**Published:** 2017-11-16

**Authors:** Wouter Hoogkamer, Shalaya Kipp, Jesse H. Frank, Emily M. Farina, Geng Luo, Rodger Kram

**Affiliations:** 1 Locomotion Lab, Department of Integrative Physiology, University of Colorado, Boulder, 354 UCB, Boulder, CO 80309-0354 USA; 2 Nike Sport Research Lab, One Bowerman Drive, Beaverton, OR 97005 USA

## Abstract

**Background:**

Reducing the energetic cost of running seems the most feasible path to a sub-2-hour marathon. Footwear mass, cushioning, and bending stiffness each affect the energetic cost of running. Recently, prototype running shoes were developed that combine a new highly compliant and resilient midsole material with a stiff embedded plate.

**Objective:**

The aim of this study was to determine if, and to what extent, these newly developed running shoes reduce the energetic cost of running compared with established marathon racing shoes.

**Methods:**

18 high-caliber athletes ran six 5-min trials (three shoes × two replicates) in prototype shoes (NP), and two established marathon shoes (NS and AB) during three separate sessions: 14, 16, and 18 km/h. We measured submaximal oxygen uptake and carbon dioxide production during minutes 3–5 and averaged energetic cost (W/kg) for the two trials in each shoe model.

**Results:**

Compared with the established racing shoes, the new shoes reduced the energetic cost of running in all 18 subjects tested. Averaged across all three velocities, the energetic cost for running in the NP shoes (16.45 ± 0.89 W/kg; mean ± SD) was 4.16 and 4.01% lower than in the NS and AB shoes, when shoe mass was matched (17.16 ± 0.92 and 17.14 ± 0.97 W/kg, respectively, both *p* < 0.001). The observed percent changes were independent of running velocity (14–18 km/h).

**Conclusion:**

The prototype shoes lowered the energetic cost of running by 4% on average. We predict that with these shoes, top athletes could run substantially faster and achieve the first sub-2-hour marathon.

## Key Points

**Table:** 

Recently, running shoes were developed that combine a new highly compliant and resilient midsole material with a stiff embedded plate.
We showed that these newly developed running shoes reduce the energetic cost of running by an average of 4% compared with established marathon racing shoes.
We predict that with these shoes, top athletes can run substantially faster and achieve the first sub-2-hour marathon.

## Introduction

Like the quest to run the first sub-4-minute mile [1], the possibility of running a sub-2-hour marathon has captivated the interest of the public, athletes, and scientists [2–4]. The world record for the 42.2 km (26.2 miles) marathon is 2:02:57 and thus a 1:59:59 time would require running 2.5% faster. Three physiological parameters generally determine and predict the running velocity that can be sustained: the maximal rate of oxygen uptake (${\dot{\text{V}}\text{O}}_{{ 2 {\text{max}}}}$), the lactate threshold, and the energetic cost of running (running economy) [5, 6]. Running economy has traditionally been defined as the rate of oxygen uptake in mL O_2_/kg/min required to run at a specified velocity. However, since oxygen uptake alone does not reflect metabolic substrate differences [7], we prefer to define running economy as the energetic cost of running at a specific velocity expressed in W/kg. Among elite distance runners with a similar ${\dot{\text{V}}\text{O}}_{{ 2 {\text{max}}}}$ and lactate threshold, a runner with a better running economy (i.e., lower energetic cost of running) can be expected to outperform runners with a higher energetic cost of running [8]. If an athlete can lower their energetic cost to run at a specified velocity, then they should be able to run faster with their existing physiological capacities [9].

Footwear mass, cushioning, and longitudinal bending stiffness each affect the energetic cost of running. Lighter running shoes reduce the energetic cost of running [10, 11], likely due to the reduced inertia for leg swing. Such energetic savings directly translate to faster performance [9]. Running barefoot might seem optimal since it involves zero shoe mass, but barefoot running is not energetically optimal because it requires greater muscular effort for cushioning the foot–ground impact [12, 13]. Experiments using special treadmills with springy or cushioned surfaces have demonstrated up to 12% energy savings [13, 14] that are attributed to two factors. First, cushioning allows a person to run with straighter legs (less knee flexion) and thus less muscular effort [14, 15]. Second, treadmill surfaces can store and return mechanical energy [14, 16, 17].

Virtually all modern running shoes have midsoles made from various foam materials that, to varying degrees, cushion impact, store and return mechanical energy. The amount of energy stored by a foam material depends on its compliance—the amount of compression that occurs when loaded with a certain force. Compliant foams are commonly described as soft. Inevitably, all foams are viscoelastic; i.e., they dissipate some energy as heat [18]. The percent of the stored mechanical energy that is returned is called resilience. Some materials/surfaces are compliant, but have low resilience (e.g., a sandy beach) and thus increase the energetic cost of running [19]. However, compliance and resilience are not mutually exclusive and new materials continue to advance shoe technology. Recently, more compliant and resilient shoe midsoles have been shown to reduce the energetic cost of running by ~ 1% [20]. Taking these observations together, theoretically, the best running shoe foam would be lightweight, highly compliant, and resilient.

Running shoes can also enhance how the human foot acts like a lever [21] to transmit the force developed by the leg muscles (e.g., the calf) to the ground so that the body is propelled upward and forward. To do so, scientists have incorporated carbon-fiber plates into the midsole, thereby increasing the longitudinal bending stiffness. Such plates can reduce the energetic cost of running by ~ 1% [22] through changes in the leverage of the ankle joint and the foot–toe joint (metatarsophalangeal joint) [22–24].

**Fig. 1:**
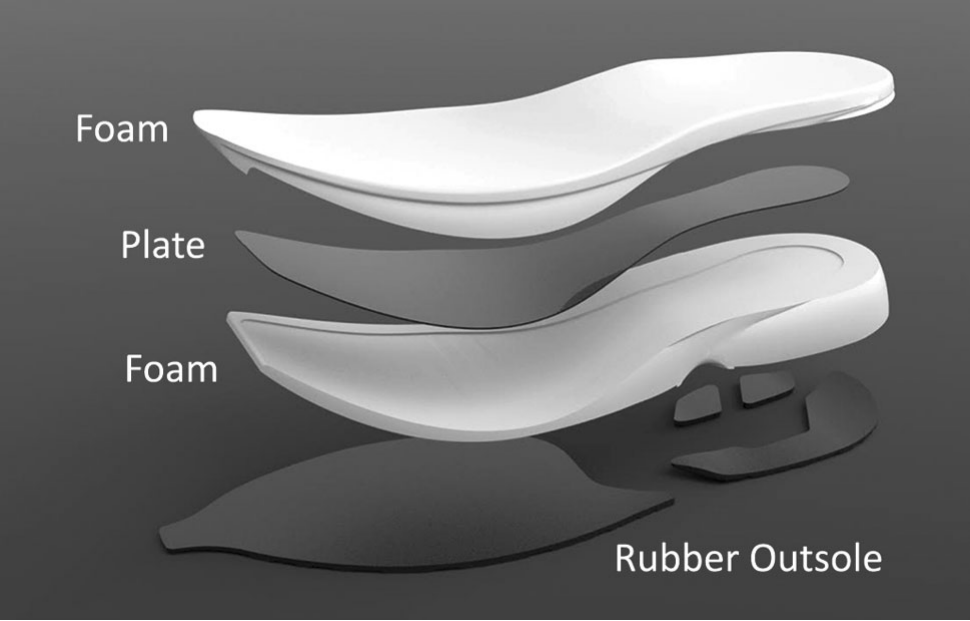
Exploded view of the Nike prototype shoe that incorporates a newly developed midsole material and a full-length carbon-fiber plate with forefoot curvature, embedded in the midsole

Recently, prototype running shoes were developed by Nike, Inc. that combine a new highly compliant and resilient midsole material with a stiff embedded plate (Fig. 1). The purpose of this study was to determine if, and to what extent, these newly developed running shoes reduce the energetic cost of running (i.e. improve running economy) compared with established marathon racing shoes. We compared both the energetics and gross biomechanics of running in the Nike prototype shoes (NP) with those of baseline marathon racing shoes, the Nike Zoom Streak 6 (NS) and the shoes used to run the official marathon world record, the adidas adizero Adios BOOST 2 (AB). The NS and AB or their predecessors were used to run the 10 fastest marathons prior to the start of this study (early April 2016).

We hypothesized that the energetic cost of running would be substantially reduced in the prototype shoes as compared with the two established marathon racing shoe models. We had no *a priori* hypotheses regarding biomechanics, but collected the data to possibly explain any energetic differences found. Furthermore, we set out to relate any potential reductions in the energetic cost of running in the prototype shoes to elite marathon running performance and the sub-2-hour marathon barrier.

## Materials and Methods

### Shoe Conditions

We compared new prototype shoes (NP, a prototype of the recently released Nike Zoom Vaporfly) to baseline marathon racing shoes, the Nike Zoom Streak 6 (NS), and the shoes that Dennis Kimetto wore when he set the current marathon world record, the adidas adizero Adios BOOST 2 (AB) (Fig. 3). We added 51 and 47 g of lead pellets to the NP and NS shoes, respectively, to equalize to the greater mass of the AB shoes (250 g for size US10). This prevented the confounding effects of shoe mass on the energetic cost of running [9–11]. To prevent excessive wear accumulation in the shoes, we used three pairs of each shoe model in size US10 and two additional pairs of AB size US9.5, because that model fits a little bigger than the Nike models. Total running use for any pair of shoes did not exceed 50 km.

### Mechanical Testing Protocol

To evaluate the relevant midsole properties, we used a custom mechanical testing method developed in the Nike Sport Research Lab. Rather than a more conventional energy-constrained impact test [25], we implemented a force-constrained mechanical testing approach [20, 26]. This method allows for more realistically quantifying of underfoot mechanical energy storage and return. We performed the shoe mechanical testing after the running tests to obviate possible cushioning inconsistencies that can arise during an initial midsole ‘break-in’ period.

**Fig. 2:**
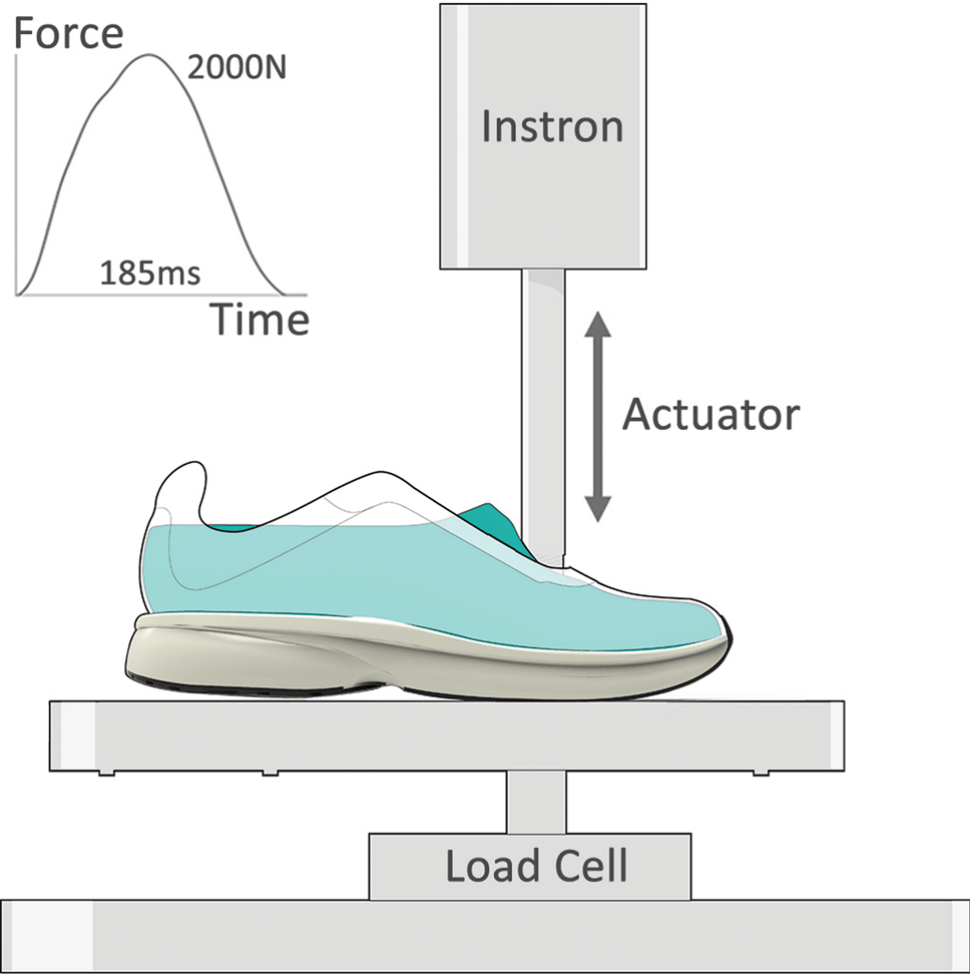
A rigid foot-form (shoe last) was mounted to the material testing machine actuator and snugly fit into a fully-constructed shoe. The actuated foot-form compressed the midsole in the vertical direction to match the displayed general time history of the vertical ground reaction force, producing insole pressure patterns similar to those recorded during running at 18 km/h

To properly execute a force-constrained mechanical test, the compression force and regional distribution of force needs to resemble that of human running. To implement this, we mounted a rigid foot-form (shoe last) to a material testing machine (Instron 8800 Series Servohydraulic System, Norwood, MA, USA) and snugly fit the foot-form into the fully constructed shoes (Fig. 2). The material testing machine compressed the midsole in the vertical direction by matching a general time history of the vertical ground reaction force measured during running. The force profile had a peak magnitude of ~ 2000 N and a contact time of ~ 185 ms, which is similar to the loading that we measured for our subjects at 18 km/h (Table 2). The foot-form shape and its material testing machine attachment location produced insole pressure patterns and magnitudes similar to those recorded during running. We calculated the amount of mechanical energy stored and returned for each shoe condition from the area under the rising (storage) and falling (return) portions of the force-deformation curves.

This custom test is limited to 1-dimensional actuation of force over a pre-defined contact region. True running force fidelity would require 3-dimensional forces, with options for different loading phases to impart load on different regions of the midsole. In addition, the way each runner interacts with a shoe can vary due to many factors including body mass, running velocity, and foot strike pattern. Though limited, this simplified testing method does provide a clean, general characterization of midsole mechanical energy storage and return capabilities in a direction relevant to the spring-mass behavior of runners [27].

**Fig. 3:**
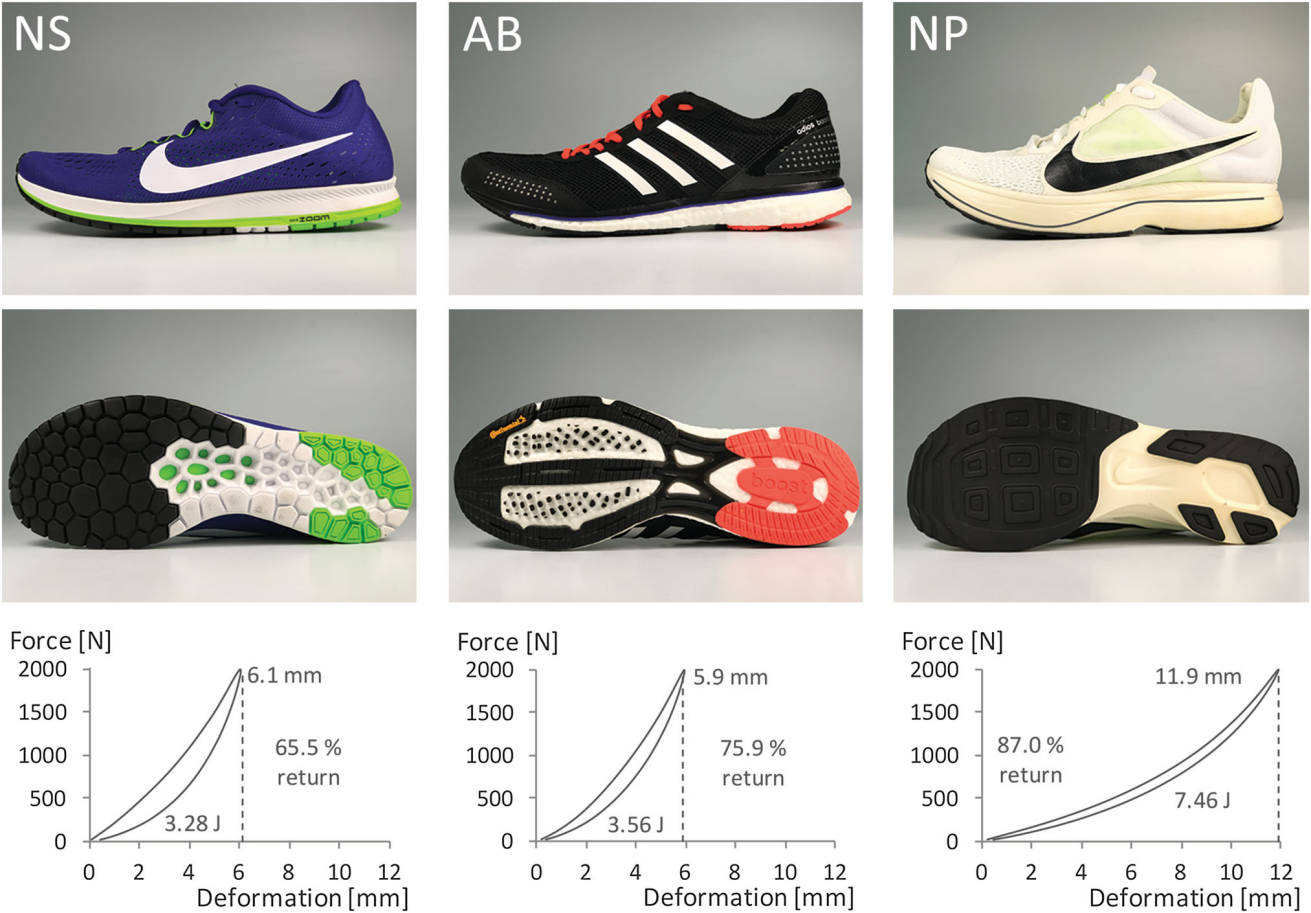
We performed mechanical testing on three marathon racing shoe models. (Top left) The Nike Zoom Streak 6 (NS) midsole comprises lightweight EVA (ethylene-vinyl acetate) foam, a rearfoot Zoom air bag, 23 mm heel height, and 15 mm forefoot height. (Top middle) The adidas adizero Adios BOOST 2 (AB) midsole comprises BOOST foam made with TPU (thermoplastic polyurethane), 23 mm heel height, and 13 mm forefoot height. (Top right) The Nike prototype (NP) midsole comprises a new ZoomX foam made with PEBA (polyether block amide), an embedded carbon fiber plate, 31 mm heel height, and 21 mm forefoot height. (Bottom) Force-deformation curves, peak deformation, and energy return metrics for each shoe during vertical midsole loading with a peak force of ~ 2000 N and contact time of ~ 185 ms (Table 2). As vertical force is applied, the shoe midsole deforms (upper trace in each graph). Then, as the shoe is unloaded, the force returns to zero as the midsole recoils (lower trace in each graph). The area between loading and unloading curves indicates the mechanical energy (J) lost as heat. The area below the lower traces represents the amount of elastic energy (J) that is returned

The mechanical testing revealed that the NP was approximately twofold more compliant than the NS and AB shoes, deforming 11.9 mm versus 6.1 and 5.9 mm, respectively (Fig. 3). The NP stored substantially more mechanical energy (area under the top trace). Furthermore, the NP shoes were more resilient (87.0% energy return) than the AB (75.9%) and NS (65.5%) shoes. Thus, combined, the NP shoes can return more than twice the amount of mechanical energy as the other shoes, which is mainly due to its substantially greater compliance rather than the greater percent resilience.

### Human Subjects

18 male (aged 23.7 ± 3.9 years, mass 64.3 ± 4.7 kg, height 177.8 ± 4.6 cm) high-caliber runners who wear men’s shoe size US10 completed the testing protocol (${\dot{\text{V}}\text{O}}_{{ 2 {\text{max}}}}$ at the local altitude ~ 1655 m: 72.1 ± 3.4 mL O_2_/kg/min, range 66.4–81.4 mL O_2_/kg/min). All had recently run a sub-31 minute 10-km race at sea level, a sub-32 minute 10-km race at the local altitude, or an equivalent performance in a different distance running event. The study was performed in accordance with the ethical standards of the Declaration of Helsinki. Ethics approval was obtained from the University of Colorado Institutional Review Board (Protocol# 15-0114). Before taking part in the study, participants provided informed written consent.

### Experimental Protocol

The study comprised four visits for each subject. Visit 1 established that subjects could run below their lactate threshold [28] at 14, 16, and 18 km/h by measuring blood lactate concentrations ([La]). During visits 2, 3, and 4, we measured the subjects’ metabolic energy consumption rates, ground reaction forces, and [La] at 14, 16, or 18 km/h while wearing each of the three shoe conditions.

Subjects presented a 24-h dietary, sleep, and training log before each visit. We strongly encouraged the subjects to replicate their diet, sleep, and training pattern for all laboratory visits. If replication was not met, we postponed the testing.

#### Visit 1

Subjects wore their own shoes to run 5-min trials at velocities of 14, 16, and 18 km/h on a level treadmill and took a 5-min break between all trials. We used a hand-held digital tachometer (Shimpo DT-107A, Electromatic Equipment Inc., Cedarhurst, NY, USA) to verify the treadmill velocities. To allow familiarization, subjects breathed through the expired-gas analysis system during this session (True One 2400, Parvo Medics, Salt Lake City, UT, USA). Within 1 min after the completion of each 5-min trial, we obtained a finger-prick blood sample for [La] determination. We analyzed the blood samples in duplicate with a YSI 2300 lactate analyzer (YSI, Yellow Springs, OH, USA). Two individuals were excluded from the study after Visit 1, reaching [La] values of 5.27 and 5.69 mmol/L at 18 km/h. The remaining 18 subjects were running at an intensity below the onset of blood lactate accumulation (OBLA), which specifies an [La] of 4 mmol/L [28], and the average [La] at 18 km/h was 2.81 ± 0.71 mmol/L.

#### Visits 2, 3, and 4

Subjects began with a 5-min warm-up trial at 14 km/h in their own shoes. Following the warm up, subjects completed six 5-min trials at one of the three velocities (14, 16, or 18 km/h, randomized) on a level force-measuring treadmill with a rigid, reinforced aluminum deck, that recorded horizontal and vertical ground reaction forces [29]. We measured the submaximal rates of oxygen consumption and carbon dioxide production during each trial using the expired-gas analysis system and calculated the rate of metabolic energy consumption over the last 2 min of each trial, using the Brockway equation [30]. In each of the six trials, subjects wore one of the three shoe conditions. In between trials, subjects took a 5-min break while they changed shoes. Note that runners mechanically adapt their biomechanics very quickly in response to changes in surface stiffness [31]. Subjects wore each shoe model twice per visit, in a mirrored order, which was counterbalanced and randomly assigned. With three shoe conditions, there were six possible shoe orders and we randomly assigned three subjects to each order. One example of a mirrored order is AB, NS, NP, NP, NS, AB. For all metrics, we averaged the two trials for each shoe condition.

During the last 30 s of each trial, we recorded high-speed video (240 frames/s, 1/1000 s shutter) using a Casio EX-FH20 camera (Casio America, Inc., Dover, NJ, USA). During the same 30 s, we recorded horizontal and vertical ground reaction forces using a National Instruments 6009-DAQ and custom-written LabView software (National Instruments, Austin, TX, USA). We low-pass filtered the ground reaction force data at 25 Hz using a recursive 4th order Butterworth digital filter and used a 30 N threshold to determine foot-strike and toe-off events. We used the video recordings to help determine the foot strike patterns of the runners during all trials (rearfoot strike vs. mid/forefoot strike). This was done by two raters (SK and JHF) independently. When the video-based classification disagreed between raters (*n* = 4), strike pattern was classified based on visual inspection of the vertical ground reaction force traces by a third rater (WH).

Following the sixth trial on each day, subjects ran an additional trial at 14 km/h in a pair of control shoes (Nike Zoom Streak LT 2). This allowed us to measure the individual day-to-day variation in energetic cost of the subjects.

Only during visit 4, after a 10-min break, the subjects completed a ${\dot{\text{V}}\text{O}}_{{ 2 {\text{max}}}}$ test on a classic Quinton 18–60 treadmill. We set the treadmill velocity to 16 km/h and increased the incline by 1% each minute until exhaustion [32]. Subjects wore their own shoes or the control shoes. We continuously measured the rate of oxygen consumption and defined ${\dot{\text{V}}\text{O}}_{{ 2 {\text{max}}}}$ as the highest 30-s mean value obtained. Our criterion for reaching ${\dot{\text{V}}\text{O}}_{{ 2 {\text{max}}}}$ was a plateau in oxygen consumption rate after an increase in incline [33].

### Statistics

We compared energetic cost, peak vertical ground reaction force, step frequency and contact time while running in the three shoe conditions over three velocities using a two-way ANOVA with repeated measures. When we observed a significant main effect for shoe, we performed Tukey’s honest significant difference post hoc analyses to determine which shoe-by-shoe comparisons differed significantly. To evaluate any potential effects of foot strike pattern, we compared energetic cost, peak vertical ground reaction force, step frequency, and contact time using a three-way ANOVA with repeated measures (shoe × velocity × strike pattern). Furthermore, we applied multiple regression analyses to evaluate potential relationships between changes in biomechanical measures and in energetic cost of running. We used a traditional level of significance (*p* < 0.05) and performed analyses with MATLAB (The MathWorks, Inc., Natick, MA, USA) and STATISTICA (Statsoft, Tulsa, OK, USA).

To estimate how much of an improvement in marathon running performance would be predicted from a specific reduction in energetic cost, we used the curvilinear relationship between running velocity and energy cost established by Tam et al. [34]. Their model was based on overground running data in top-level Kenyan marathon runners:

${\dot{\text{V}}\text{O}}_{{2}}$ (mL O_2_/kg/min) = 5.7 + 9.8158 V + 0.0537 V^3^

with velocity (V) in m/s.

## Results

**Fig. 4:**
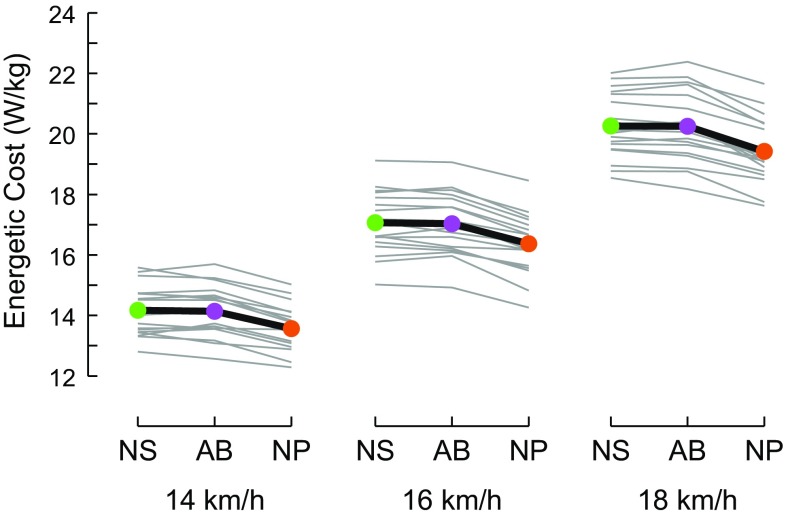
Over the three velocities tested, runners in the NP shoes used an average of 4.16% less metabolic energy than the NS shoes and 4.01% less than in the AB shoes (both *p* < 0.001). The AB and NS shoes were similar (*p* = 0.34). Values are the gross energetic cost of running. *NS* Nike Zoom Streak 6, *AB* adidas adizero Adios BOOST 2, *NP* Nike prototype

The prototype shoes substantially lowered the energetic cost of running by 4% on average. Notably, at all three running velocities, energetic cost was lower in NP for each and every subject compared with both NS and AB (Fig. 4). Averaged across all three velocities, the energetic cost for running in the NP shoes (16.45 ± 0.89 W/kg; mean ± SD) was 4.16 and 4.01% lower than in the NS and AB shoes (17.16 ± 0.92 and 17.14 ± 0.97 W/kg, respectively, both *p* < 0.001). The NS and AB shoes were similar (*p* = 0.34). The percent differences between shoes were similar at the three running velocities (all *p* > 0.56). Among the 18 subjects, the mean difference in energetic cost over the three velocities between the NP and NS shoes ranged from − 1.59 to − 6.26% and from − 1.97 to − 6.08% for NP versus AB, indicating considerable inter-individual variation in the amount of energetic saving the NP shoes provided. For reference, rates of oxygen uptake, energetic cost of transport, and the oxygen cost of transport for each of the three shoe models at all three velocities are listed in Table 1.

**Table 1:** Energetic costs, rates of oxygen uptake (${\dot{\text{V}}\text{O}}_{2}$), energetics cost of transport (ECOT) and oxygen costs of transport (O_2_COT) for each of the three shoe models at all three speeds

	14 km/h			16 km/h			18 km/h		
	NS	AB	NP	NS	AB	NP	NS	AB	NP
Energetic cost (W/kg)	14.17 ± 0.82	14.13 ± 0.84	13.57 ± 0.76	17.07 ± 1.02	17.03 ± 1.02	16.36 ± 0.99	20.26 ± 1.06	20.25 ± 1.18	19.42 ± 1.08
VO_2_ (mL O_2_/kg/min)	41.97 ± 2.39	41.87 ± 2.45	40.24 ± 2.19	50.30 ± 2.91	50.19 ± 2.92	48.27 ± 2.87	59.62 ± 3.08	59.57 ± 3.40	57.26 ± 3.10
ECOT (J/kg/m)	60.72 ± 3.52	60.57 ± 3.59	58.15 ± 3.25	64.00 ± 3.83	63.85 ± 3.84	61.36 ± 3.71	67.52 ± 3.55	67.49 ± 3.94	64.72 ± 3.60
O_2_COT (mL O_2_/kg/km)	179.9 ± 10.3	179.4 ± 2.5	172.5 ± 9.4	188.6 ± 10.9	188.2 ± 10.9	181.0 ± 10.6	198.7 ± 10.3	198.6 ± 11.3	190.9 ± 10.4

*NS* Nike Zoom Streak 6, *AB* adidas adizero Adios BOOST 2, *NP* Nike prototype

Values presented are mean ± SD

Respiratory exchange ratios (${\dot{\text{V}}\text{CO}}_{2}$/${\dot{\text{V}}\text{O}}_{2}$) remained < 0.9 for all trials and [La] values after six trials were < 3 mmol/L for all velocities, but we did detect a slight slow component in our recordings of oxygen consumption. Across all conditions, the rate of oxygen consumption averaged 1.0% greater during minute 5 versus minute 4 (*p* < 0.001). This was independent of shoe condition and running velocity, and all the differences between conditions were consistent for both minutes (all *p* > 0.39). For the control shoes at 14 km/h, the mean day-to-day difference in energetic cost was 2.7%, the mean minimum day-to-day difference was 1.0% and the mean maximum day-to-day difference was 4.3%. Recall that we randomized and counterbalanced the order in which subjects ran at each of the three velocities (14, 16, 18 km/h) to balance out this day-to-day variation. Since subjects wore each pair of shoes twice per visit, in a mirrored order, we could quantify within-day variation. The mean absolute variation over all running velocities and shoe conditions was 1.7%.

**Fig. 5:**
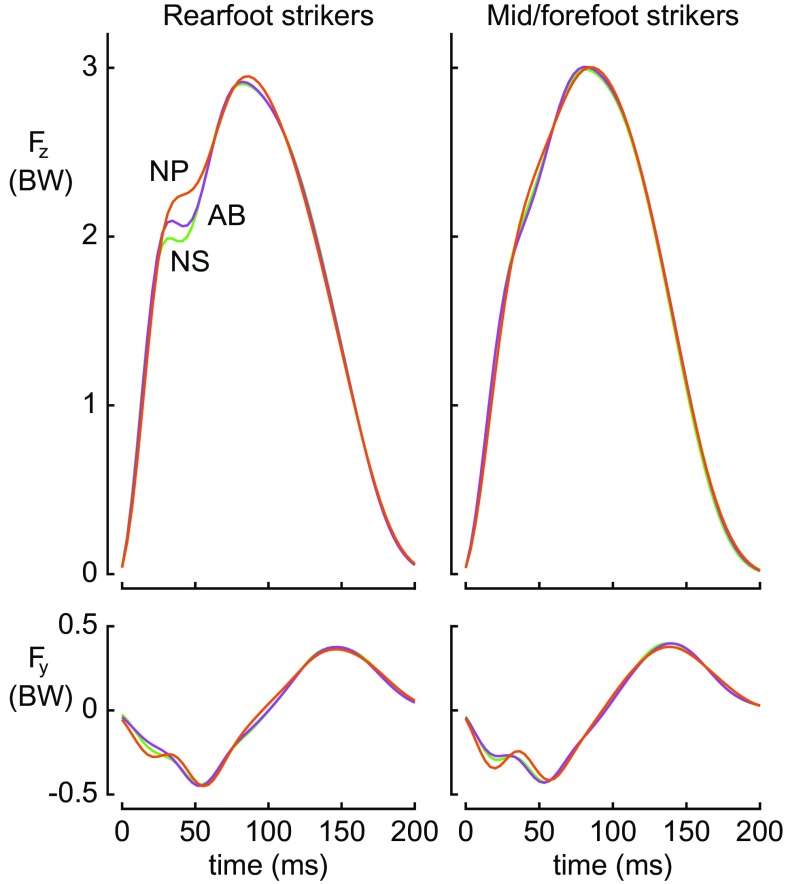
Average vertical (*F*_z_; top) and anterior–posterior ground reaction force traces (*F*_y_; bottom) in the three different shoe models for runners with rearfoot strike pattern (*n* = 8) (left) and midfoot or forefoot strike pattern (*n* = 10) (right) during the 16-km/h trials. Force traces are normalized to body weight (*BW*). Initial impact and active *F*_z_ peaks were greater for the rearfoot strikers in the NP shoes. *F*_z_ recordings for mid/forefoot strikers were similar in the three shoes. *NS* Nike Zoom Streak 6, *AB* adidas adizero Adios BOOST 2, *NP* Nike prototype

While running in the NP shoes, the subjects generally ran with slightly greater peak vertical ground reaction forces, slower step frequencies, and longer contact times than in the control shoes (Fig. 5; Table 2). Peak vertical ground reaction force (*F*_z_) was 1.1% greater in the NP shoes than in the NS shoes (*p* = 0.002) and increased at faster running velocities in all shoes (all *p* < 0.001). Step frequency was 0.8 and 0.6% slower in the NP shoes than in the NS and AB shoes, respectively; that is, slightly longer steps in NP (both *p* < 0.001). Step frequency increased at the faster running velocities in all shoes (all *p* < 0.001). Contact time was slightly (0.6%) longer in the NP shoes than in the NS shoes (*p* = 0.020) and decreased at faster running velocities in all shoes (all *p* < 0.001). Together, the percent changes in peak *F*_z_, step frequency, and contact time explained 20% of the variance in the reductions in energetic cost between NS and NP (*p* = 0.009). Peak *F*_z_ was the only individual biomechanical factor contributing significantly and energetic savings were paradoxically correlated to *increases* in peak *F*_z_. The changes in energetic cost between AB and NP or between NS and AB were not significantly correlated to changes in biomechanical measures (*p* = 0.095 and *p* = 0.8, respectively).

**Table 2:** While running in the NP shoe, the subjects generally ran with slightly greater peak vertical ground reaction forces, slower step frequencies and longer contact times than in the control shoes

	NS	AB	NP
Peak *F*_z_ (BW)	*		
14 km/h	2.88 ± 0.19	2.89 ± 0.20	2.92 ± 0.20
16 km/h	2.98 ± 0.19	3.00 ± 0.19	3.00 ± 0.17
18 km/h	3.11 ± 0.18	3.14 ± 0.18	3.13 ± 0.18
Step frequency (Hz)	*	*	
14 km/h	2.90 ± 0.14	2.89 ± 0.15	2.87 ± 0.14
16 km/h	2.97 ± 0.15	2.97 ± 0.16	2.96 ± 0.15
18 km/h	3.05 ± 0.16	3.04 ± 0.16	3.02 ± 0.16
Contact time (ms)	*		
14 km/h	212 ± 8	212 ± 8	213 ± 8
16 km/h	197 ± 8	196 ± 7	197 ± 7
18 km/h	180 ± 5	181 ± 5	182 ± 5

Peak vertical ground reaction forces (*F*_z_) are normalized to body weight (*BW*)

*NS* Nike Zoom Streak 6, *AB* adidas adizero Adios BOOST 2, *NP* Nike prototype

Values presented are mean ± SD

*Indicates significantly different from NP shoes across running velocities

Although we did not set out to evaluate the foot strike pattern interaction on the energetic cost differences between shoes, our sample of runners did allow for such an analysis. Eight of our subjects landed on their heels and ten landed on their mid/forefoot. Overall, the energetic cost of running was not different between rearfoot strikers and mid/forefoot strikers (*p* = 0.9; Table 3). However, a shoe × foot strike pattern interaction effect (*p* = 0.0502) suggests that the savings in the NP shoes were likely somewhat greater for rearfoot strikers (NP vs. NS: 4.78%; NP vs. AB: 4.63%) than for mid/forefoot strikers (3.67 and 3.50%, respectively). We did not observe significant shoe × foot strike interactions for any of the biomechanical parameters, but rearfoot strikers ran with longer contact times than mid/forefoot strikers (*p* = 0.001; Table 3).

**Table 3:** Energetic costs and biomechanics variables for each of the three shoe models at all three speeds, separated by foot strike type

	14 km/h			16 km/h			18 km/h		
	NS	AB	NP	NS	AB	NP	NS	AB	NP
Energetic cost (W/kg) rearfoot strike	14.21 ± 0.91	14.17 ± 0.81	13.54 ± 0.82	17.09 ± 0.96	17.01 ± 0.98	16.25 ± 0.95	20.40 ± 1.27	20.44 ± 1.41	19.43 ± 1.31
Energetic cost (W/kg) mid/forefoot strike	14.13 ± 0.79	14.10 ± 0.90	13.59 ± 0.75	17.05 ± 1.12	17.04 ± 1.11	16.45 ± 1.06	20.15 ± 0.92	20.10 ± 1.01	19.40 ± 0.93
Peak *F*_z_ (BW) rearfoot strike	2.81 ± 0.16	2.82 ± 0.16	2.85 ± 0.16	2.93 ± 0.13	2.95 ± 0.12	2.97 ± 0.12	3.05 ± 0.12	3.09 ± 0.14	3.09 ± 0.13
Peak *F*_z_ (BW) mid/forefoot strike	2.93 ± 0.21	2.94 ± 0.24	2.98 ± 0.24	3.02 ± 0.23	3.04 ± 0.23	3.03 ± 0.21	3.15 ± 0.23	3.17 ± 0.21	3.17 ± 0.21
Step frequency (Hz) rearfoot strike	2.86 ± 0.14	2.85 ± 0.13	2.84 ± 0.14	2.93 ± 0.13	2.92 ± 0.13	2.89 ± 0.13	3.00 ± 0.13	2.99 ± 0.13	2.96 ± 0.12
Step frequency (Hz) mid/forefoot strike	2.93 ± 0.15	2.93 ± 0.16	2.90 ± 0.16	3.02 ± 0.16	3.01 ± 0.18	3.01 ± 0.16	3.09 ± 0.18	3.08 ± 0.18	3.07 ± 0.17
Contact time (ms) rearfoot strike	218 ± 3	218 ± 5	220 ± 2	203 ± 5	201 ± 5	202 ± 4	184 ± 3	185 ± 4	186 ± 3
Contact time (ms) mid/forefoot strike	207 ± 8	208 ± 8	208 ± 7	192 ± 8	193 ± 7	193 ± 7	177 ± 5	178 ± 5	178 ± 4

*NS* Nike Zoom Streak 6, *AB* adidas adizero Adios BOOST 2, *NP* Nike prototype, *F*_z_ vertical ground reaction force, *BW* body weight

Values presented are mean ± SD

## Discussion

The prototype shoes substantially lowered the energetic cost of running by 4% on average. Shoe properties such as mass, midsole compliance, resilience, and longitudinal bending stiffness have all been shown to affect the energetic cost of running [20, 22]. However, reported energetic savings due to running shoe properties are typically trivial to small [35]. For every 100 g of added mass per shoe, the energetic cost of running increases by ~ 1.0%. To prevent the confounding effects of shoe mass on the energetic cost of running [9–11], we added 51 and 47 g of lead pellets to the NP and NS shoes, respectively, to equalize to the greater mass of the AB shoes. This suggests that unweighted NP shoes would likely save an average of ~ 4.4% versus AB; assuming a conservative 0.8% savings per 100 g of shoe mass [9, 10]. Midsole air bag and BOOST foam (made with thermoplastic polyurethane) cushioning have each been shown to reduce the energetic cost of running by 1–2.8% [12, 36] or 1.1% [20], respectively, as compared with conventional EVA (ethylene-vinyl acetate) foam. Here, we compared the NP shoes to two established marathon racing shoe models, which incorporate either an air bag or BOOST foam, and find an additional 4% savings with the new shoes.

While the observed differences in energetic cost of running between shoe conditions were as substantial as 4%, the differences in our gross biomechanical measures (i.e., peak *F*_z_, step frequency, and contact time) were on the order of only 1% (Table 2). Subjects ran with slower step frequency, taking longer steps in the NP shoes. This is in line with the observed higher peak *F*_z_ and longer contact time in the NP shoes compared with the NS shoes. However, differences of < 1% in these variables seem too small to have a substantial influence on energetic cost of running. This was confirmed by the multiple regression analyses between the percent changes in each of the biomechanical measures and in the energetic cost. A significant correlation was only observed for the differences between NS and NP, with changes in biomechanics explaining < 20% of the energetic differences. Further, the differences in peak *F*_z_ and in contact time were only significant between NP and NS, not between NP and AB, even though energetic savings for NP were similar to those for NS and AB.

Although gross measures of biomechanics showed little differences between the different shoes, a biomechanical explanation for the energetic savings is important to consider. When running on compliant surfaces, people maintain their center of mass mechanics by reducing knee flexion during the stance period, which increases leg stiffness [31]. This improves the mechanical advantage of the muscles acting around the joints, which reduces the energetic cost of body weight support [14, 37]. This same mechanism likely contributes to the energy savings of the very compliant NP shoes. However, we did not record joint kinematics in the present study and thus cannot yet quantify any differences in peak knee flexion during stance in the different shoes.

For now, the elastic properties of the NP shoes provide the best explanation for the metabolic energy savings. Our mechanical testing quantified that the NP shoes returned 7.46 J of mechanical energy per step versus 3.38 and 3.56 J for the NS and AB shoes, respectively (Fig. 3). The greater mechanical energy return in the NP shoes is mainly due to its substantially greater compliance rather than the greater percent resilience. For context, the arch of the human foot and Achilles tendon return 17 and 35 J of stored energy, respectively, during running at 16.2 km/h [38]. Other ligaments and tendons of the leg store and return additional energy [39, 40]. Thus, regardless of the shoes worn, in human running, the vast majority of the mechanical energy storage and return occurs within our natural biological structures. However, to operate the tendons as springs, the muscles that connect tendons to bones must actively contract, which consumes metabolic energy [41]. In contrast, running shoes with elastic midsoles and stiffening plates may reduce rather than require the generation of muscular force.

How much of an improvement in running performance would be predicted from a 4% reduction in energetic cost? Hoogkamer et al. [9] established that percent changes in the energetic cost of running due to altered shoe mass translate to similar percent changes in 3000-m running performance, when both are evaluated at the same running velocity. But, as recently summarized by Hoogkamer et al. [3], the energetic cost of overground running increases curvilinearly with velocity, due in part to air resistance. Such curvilinearity implies that a 4% average energetic savings observed should translate to ~ 3.4% improvement in running velocity at marathon world record pace (20.59 km/h) [3, 34]. Consistent with that calculation, in the two years leading up to her amazing world record in the women’s marathon in 2003, directed training allowed Paula Radcliffe to reduce her energetic cost of running at 16 km/h by 2.8% and marathon performance by 2.4% [42]. An acute 3.4% improvement in the marathon world record would be historic. For example, it took nearly 29 years for the men’s marathon record to be reduced by ~ 3.4% to the current 2:02:57, and not since 1952 has the men’s marathon record been broken by more than 3.4% in one race.

Note that we empirically compared the shoes up to a running velocity of 18 km/h, about 13% slower than the average marathon world record velocity. It was challenging to recruit 18 runners who could sustain 18 km/h below lactate threshold and also fit the available size US10 prototypes. Therefore, we tested a range of velocities to determine if any energy savings were dependent on running velocity. Over the tested velocity range of 14–18 km/h, the percent savings were constant. The energetic cost of running for elite marathon runners is likely lower than in our high-caliber, sub-elite runners [43, 44], and the energetic cost of running may slowly increase over the duration of a marathon [45], due to slow component increases in oxygen uptake kinetics [46] and muscle damage [47], as compared with the energy cost values we observed. How the 4% savings we observed, interact with all these variables remains to be determined.

In conclusion, the new running shoes described herein provide 4% energetic savings. Our extrapolations suggest that with these shoes the technology is in place to break the 2-h marathon barrier. Now, it is up to the athletes to make it happen.
